# Enhancing the incorporation of the patient’s voice in drug development and evaluation

**DOI:** 10.1186/s40900-018-0093-3

**Published:** 2018-04-02

**Authors:** Meghana Chalasani, Pujita Vaidya, Theresa Mullin

**Affiliations:** 0000 0001 2243 3366 grid.417587.8 U.S. Food and Drug Administration, WO51, Room 1146, 10903 New Hampshire Avenue, Silver Spring, MD 20993-0002 USA

**Keywords:** Patient-focused drug development, Patient engagement, Patient perspectives, FDA, Benefit-risk

## Abstract

**Plain English summary:**

People living with a condition are uniquely positioned to inform the understanding of the therapeutic context for drug development and evaluation. In 2012, the U.S. Food and Drug Administration (FDA) established the Patient-Focused Drug Development (PFDD) initiative to more systematically obtain the patient perspective on specific diseases and their currently available treatments. PFDD meetings are unique among FDA public meetings, with a format designed to engage patients and elicit their perspectives on two topic areas: (1) the most significant symptoms of their condition and the impact of the condition on daily life; and, (2) their current approaches to treatment. FDA has conducted 24 disease-specific PFDD meetings to date. The lessons learned from PFDD meetings range from experiences common across rare diseases to more disease specific experiences that matter most to patients. FDA recognizes that FDA-led PFDD meetings alone cannot address the gaps in information on the patient perspective. Patient-focused drug development is an ongoing effort and FDA looks forward to the next steps in advancing the science and the utilization of patient input throughout drug development and evaluation.

**Abstract:**

The U.S. Food and Drug Administration (FDA) has multiple mechanisms for its regulators and staff to interact with patients -- but none quite like its novel Patient-Focused Drug Development (PFDD) initiative. FDA established the PFDD initiative to more systematically obtain the patient perspective on specific diseases and their currently available treatments. Since the initiative’s inception in 2012, FDA has held 24 PFDD meetings, covering a range of disease areas and hearing directly from thousands of patients and caregivers. FDA’s PFDD meetings have also provided key stakeholders, including patient advocates, researchers, drug developers, healthcare providers, and other government officials, an opportunity to hear the patient’s voice. The lessons learned include but are not limited to specific experiences that matter most to patients, patient perspectives on meaningful treatment benefits and how patients want to be engaged in the drug development process. FDA recognizes that FDA-led PFDD meetings alone cannot address the gaps in information on the patient perspective. Further enhancing the incorporation of the patient’s voice in drug development and evaluation continues to be a priority for FDA.

## Background: FDA-led Patient-Focused Drug Development (PFDD) meetings

People living with a condition are uniquely positioned to inform the understanding of the therapeutic context for drug development and evaluation. They can articulate the daily impact of living with the symptoms of their condition, share in detail their experiences with currently available treatments, and highlight which factors they take into account when making decisions about a course of treatment. Previously, FDA mechanisms to directly obtain patient perspectives were typically limited to discussions related to specific medical product applications under review, such as through an advisory committee meeting [1]. FDA recognized that there was a need for more systematic ways of gathering patient perspectives on their condition and treatment options, also known as patient experience data. As part of FDA’s commitments under the fifth authorization of the Prescription Drug User Fee Act (PDUFA V), FDA established the Patient-Focused Drug Development (PFDD) initiative which aims to more systematically obtain the patient perspective on specific diseases and their currently available treatments.

**Fig. 1:**
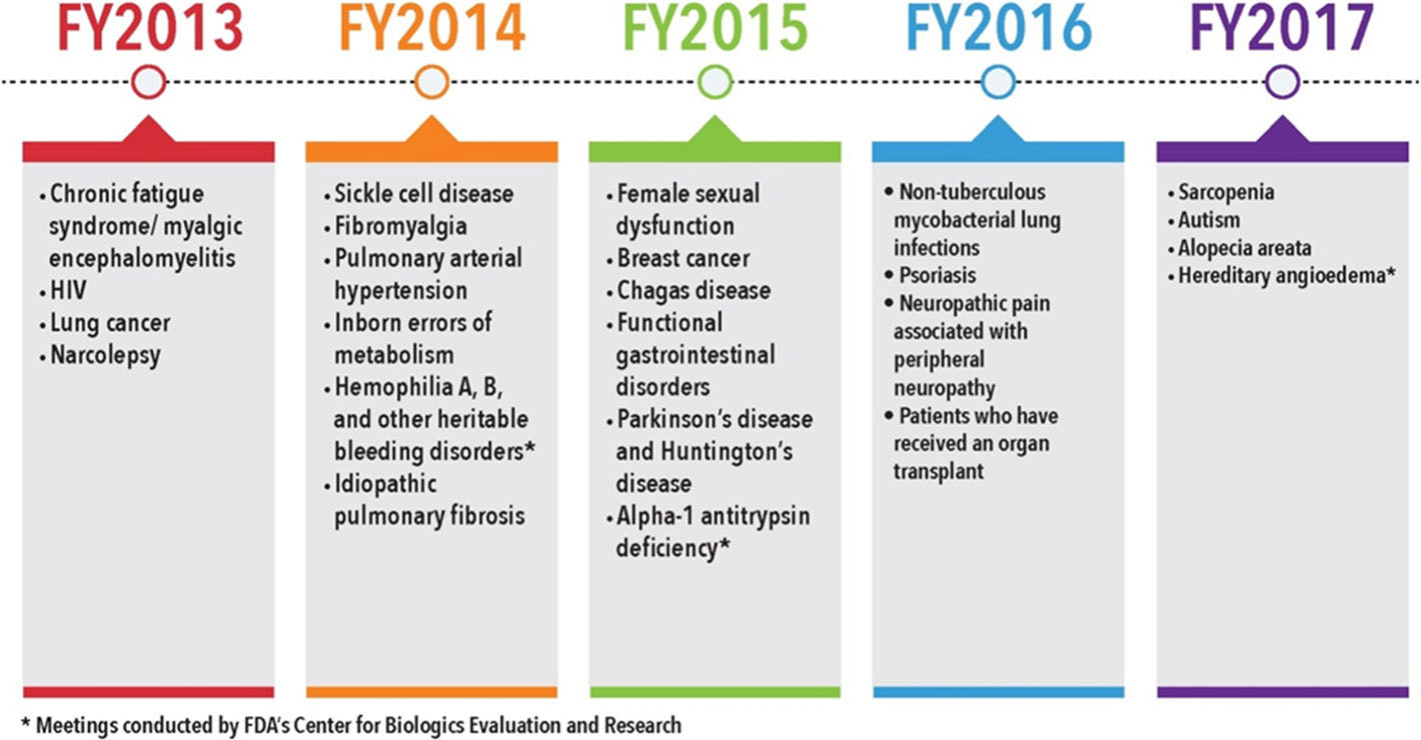
PDUFA V Patient-Focused Drug Development Public Meeting Disease Areas

During the five-year period of PDUFA V (Fiscal Year (FY) 2013–2017), FDA’s Center for Drug Evaluation and Research (CDER) and Center for Biologics Evaluation and Research (CBER) convened PFDD meetings on specific disease areas (Fig. 1). In selecting the set of disease areas of focus for the PFDD meetings, FDA proposed the following selection criteria:

- Disease areas that are chronic, symptomatic, or affect functioning and activities of daily living
- Disease areas for which aspects of the disease are not formally captured in clinical trials
- Disease areas for which there are currently no therapies or very few therapies, or the available therapies do not directly affect how a patient feels, functions, or survives
- Disease areas that have a severe impact on identifiable subpopulations (such as children or the elderly)

In September 2012, FDA published a public notice announcing the selection criteria and identified 39 disease areas as potential candidates. FDA obtained comments on the proposed criteria and potential disease areas through a public docket and a public meeting. A docket is a repository through which the public can submit electronic and written comments on specific topics to U.S. federal agencies such as FDA [2]. Almost 4500 comments addressing over 90 disease areas were submitted by patients, caregivers, patient advocates and advocacy groups, healthcare providers, professional societies, scientific and academic experts, drug developers, and others. The majority of comments were submitted by individual patients. Input from the public was particularly helpful for FDA in better understanding the aspects of diseases that may not currently be formally measured in clinical trials as well as cases where available therapies do not directly address the aspects of disease that matter most to patients. In April 2013, FDA published a public notice announcing the disease areas for meetings in FYs 2013–2015, the first three years of the five-year PDUFA V time frame [3]. In selecting the set of disease areas, FDA carefully considered the public input received and the perspectives of review divisions at FDA. Review divisions at FDA (e.g., Division of Neurology Products, Division of Psychiatry Products, etc.) provide regulatory oversight during drug development, make decisions regarding marketing approval for new drugs, and provide guidance to regulated industry on clinical, scientific and regulatory matters.

In October 2014, FDA initiated a second public process for determining the disease areas for FY 2016–2017 by publishing a public notice identifying 16 disease areas as potential candidates and invited public comment on the preliminary list and on disease areas that were not listed. Following publication of the notice, almost 2700 comments, addressing over 50 disease areas, were submitted by patients, caregivers, patient advocates and advocacy groups, healthcare providers, professional societies, scientific and academic experts, drug developers, and others. Similar to the first round, majority of comments were submitted by individual patients. In July 2015, FDA published a public notice announcing the selection of eight disease areas for meetings in FY 2016–2017 [3].

Early in the development of the PFDD initiative, FDA periodically convened meetings with patient stakeholders to discuss issues related to the process and execution of the initiative, including how to approach issues when patient stakeholders for the same disease area have different and potentially conflicting views and how to support engagement of patients in disease areas for which no formal advocacy organizations exist. These meetings provided valuable insight into how best to engage with patients when conducting the subsequent PFDD meetings, including critical input in the development of the meeting format and discussion questions [4].

## Overview of PFDD meeting topics and format

PFDD meetings are unique among FDA public meetings, with a format designed to engage patients and elicit their perspectives on two topic areas: (1) the most significant symptoms of their condition and the impact of the condition on daily life; and, (2) their current approaches to treatment. Each PFDD meeting is tailored to the needs of the specific disease area taking into consideration the current state of drug development for the disease, the needs of the patient population, and specific interests of the FDA review division.

For each topic, a panel comprised of three to five patients, caregivers, and patient representatives initiates the dialogue by sharing experiences of living with the condition. Panel comments are followed by a semi-structured, large-group facilitated discussion that encourages participation from other patients, caregivers and patient representatives attending in-person and on the webcast. For Topic 1, participants are typically asked to describe how their symptoms manifest, which symptoms related to their condition have the most significant impact on their daily lives, and how their condition has changed over time. For Topic 2, participants are asked to describe their current treatment regimen, how their treatment regimen may have changed over time, which symptoms related to their condition are not improved with their treatment regimen and any other treatment downsides. Participants are also asked to identify specifically what they would look for in an “ideal treatment.” In some cases, Topic 2 discussions have also delved further into understanding participants’ decision-making process when considering a new treatment and other topic areas such as meaningful benefit and considerations for clinical trial participation.

The discussion is moderated by an FDA facilitator with participation of a panel of FDA reviewers who listen throughout the meeting and may ask follow-up questions. FDA encourages patient advocates, researchers, drug developers, healthcare providers and other government officials to attend PFDD meetings; however, FDA asks that they remain silent during the discussions since the meetings are a platform to hear directly from patients, caregivers and patient representatives. Participants who join the meeting via live webcast are also able to submit comments throughout the discussion. In-person and web participants are periodically invited to respond to polling questions, which help characterize the demographic makeup of the participants, and provide a sense of the variation and distribution of participants perspectives on a given topic. The polling questions are carefully drafted prior to each PFDD meeting by the FDA meeting planning committee; they are not intended to be a scientific survey tool, but rather a facilitation tool to help aid the discussion. Each meeting also includes a public comment session to provide an opportunity for anyone, including patient advocates, researchers, drug developers, healthcare providers, and other government officials, in the audience to briefly provide a comment. To supplement what is heard during the meeting, stakeholders including those who attended the meeting and those who may not have attended can submit their comments to FDA through a public docket for up to 60 days after the meeting.

Of the meetings conducted to date, typically 100–150 participants attend in-person, including approximately 30 to 80 patients, caregivers and patient representatives. In addition, there are typically 100–300 participants on the live webcast. Although participants in a meeting may not be statistically representative of the population living with the condition, the discussion typically reflects a range of experiences with symptoms and treatments associated with the condition. FDA also receives public comment submissions through a public docket for each meeting (with submissions ranging from five to 400 in number), from numerous stakeholders including patients, caregivers, patient representatives, patient advocates, researchers, drug developers and healthcare providers. A majority of these comments are submitted by patients or their caregivers. In addition to individual comments, patient organizations typically conduct and submit patient surveys that further examine patient perspectives on significant symptoms, impact of disease on quality of life and/or treatment options. The submitted surveys vary in survey type, participant demographics, and sample size.

## Lessons learned

Having conducted 24 disease-specific PFDD meetings under the now-completed PDUFA V initiative, FDA is grateful to the patients, caregivers and patient representatives who have so thoughtfully, generously, and often courageously shared their personal stories of living with their conditions. The patient input generated through these meetings has helped strengthen FDA’s understanding of the burden of disease on these patients and their families, and the limitations as well as the benefits of treatments currently used to manage the condition and its symptoms. Following each meeting, FDA posts a full meeting transcript and webcast recording on its public website [3]. FDA also produces a *Voice of the Patient* summary report that captures participants’ perspectives in their own words, using the meeting transcript, webcast recording, webcast comments and docket comments as sources of information [5]. These documents constitute a valuable resource that provides important patient context for FDA staff when subsequently advising sponsors on their drug development programs and when assessing products under review for marketing approval.

The input obtained from the PFDD meetings can also be of value to the drug development process more broadly, for example, helping to identify areas of unmet need in treatment of the patient population and development and qualification of new outcome measures in clinical trials. PFDD meetings have also informed the development of draft regulatory guidance to industry on drug development in a given disease area, the planning of follow-up technical workshops, and identification of patient representatives to serve on FDA advisory committees.

The lessons learned from PFDD meetings range from experiences common across rare diseases to more disease specific experiences that matter most to patients. Despite the broad range of patient experiences across disease areas, FDA has identified several key learnings from PFDD meetings, including the following examples:

### Patients are experts in what it is like to live with their disease or condition and use of available treatments

In the meetings, patients have clearly identified their most significant symptoms due to their condition (e.g., pain, fatigue, shortness of breath) and described how those symptoms have substantial impact on their daily life, including physical, social, and/or cognitive impact. Patients have also articulated their perspectives on benefits they experience from an existing treatment, discussed downsides of the treatments, and what they would look for in an ideal treatment based on their experiences from existing ones (e.g., fewer or no side effects, less frequent administration).

### Patients want their experience described with the words that they use to best describe how it feels

At the chronic fatigue syndrome and myalgic enceophalomyelitis (CFS/ME) PFDD meeting, several participants described their experience with the clinical characterization of acute, debilitating post-exertional malaise. Participants stated that the term “malaise” was inaccurate and they believed that it should be more aptly termed as “crash” or “collapse.” By narrating their experiences in their own words, patients are giving drug developers, FDA staff and others more salient descriptions that reflect their experience with their condition [6].

### Patients can best identify and articulate what is most important to them regarding treatment benefit

During the psoriasis PFDD meeting, participants identified reduced scaling, flaking and itching as benefits they would consider to be the most meaningful when considering a new treatment for psoriasis. Participants also stressed the need to enhance the treatment armamentarium, given current challenges with variability in effectiveness, access to available treatments, and uncertainty regarding long-term effects of available treatments. Several participants commented on the need to advance treatments that are not immunosuppressive as well [7].

### Patients’ “chief complaints” may not be factored explicitly into drug development plans

For example, repetitive movements, commonly known as “stimming,” are highlighted as a significant symptom of autism and used as an endpoint for developing treatments for autism. However, participants at the autism PFDD meeting stated that stimming is not their most significant symptom. A few participants shared that stimming is actually a helpful coping mechanism for them. Participants indicated that communication difficulties, such as delayed speech, repeating phrases, absence of facial expressions or inability to understand nonverbal communication or gestures, is a more significant symptom of autism and should be incorporated into future drug development plans for autism [8].

### Patients want to be as active as possible in the work to develop and evaluate new treatments

For example, in preparation for the narcolepsy PFDD meeting, various advocacy organizations coordinated their initiatives to create a new coalition specifically to prepare patients and caregivers. Similarly, for other PFDD meetings, patient organizations have helped with outreach efforts, organized transportation and held pre-meeting get-togethers or webinars to prepare participants on how to effectively engage with FDA. Most importantly, for each PFDD meeting, patients and caregivers travel at their own cost and willingly share their personal experiences. The strength and determination they have continuously demonstrated is inspiring. Patients at PFDD meetings have also expressed their strong interest in participating in clinical trials, and often highlight that their interest stems not only from their desire to see new and better treatments for themselves, but also for future generations.

## Conclusion: Further enhancing the patient voice in drug development and evaluation

Incorporating the patient voice in drug development has been and continues to be a priority for FDA and the agency has recognized that there are many more disease areas than could be addressed with the FDA’s limited meeting space and staff resources. To help expand the benefits of FDA’s PFDD initiative, FDA initiated a new “externally-led” PFDD meeting option and now welcomes patient organizations to identify and organize patient-focused collaborations to generate public input on other disease areas using the process established through FDA’s PFDD initiative as a model [9].

In addition to exploring new avenues of engaging with patients, FDA is looking to take full advantage of opportunities within existing endeavors. For example, FDA is incorporating an increased patient focus in other planned FDA scientific meetings by integrating patient elicitation methods used effectively in FDA’s PFDD meetings to facilitate a discussion among patient stakeholders.

FDA also recognizes the need to provide guidance to stakeholders interested in pursuing the development of tools to collect patient perspectives, also known as patient experience data, for a specific disease. FDA will work to advance the science and provide this guidance over the coming years to implement provisions of the 21st Century Cures Act [10–12] enacted into law in December 2016 and to fulfill FDA commitments under the sixth authorization of PDUFA (PDUFA VI) [12, 13]. The primary purpose of this guidance is to provide information and direction to external stakeholders regarding what work FDA would expect to be done to bridge from important early-stage meetings to gain patients’ narrative perspectives on the clinical context, like PFDD meetings, to development and use of methodologically-sound data collection tools in clinical trials. Patient-focused drug development is an ongoing effort and FDA looks forward to the next steps in advancing the science and the utilization of patient input throughout drug development and drug evaluation [12].

## Abbreviations

FDA: U.S. Food and Drug Administration
PDUFA V: Fifth authorization of the Prescription Drug User Fee Act
PDUFA VI: Sixth authorization of the Prescription Drug User Fee Act
PDUFA: Prescription Drug User Fee Act
PFDD: Patient-Focused Drug Development
